# North Carolina Dental Society

**Published:** 1857-01

**Authors:** 


*North Carolina Dental Society.*—A meeting of the alumni, located in this State, of the Dental Colleges, was held in Raleigh, October 16th, 1856, for the purpose of consultation upon the means best adapted to advance the science, and the common interests and honor of the profession.

The meeting came to order by the appointment of a temporary president and secretary.

On motion of Dr. Gregg, the chairman appointed a committee, consisting of Drs. Howlet, Scott and Gregg, to draft resolutions for the organization and government of a State Society.

The meeting then adjourned to meet next morning at 9 o'clock.

*Friday Morning, October 17th.*

Met agreeably to adjournment.

Members present were Drs. W. F. Bason, T. W. Howlet, D. P. Gregg, R. P. Bessent, R. Scott, West Harris and D. W. C. Benbow.

After the reading of the minutes of last meeting, the committee reported their resolutions, which were unanimously adopted.

On motion of Dr. Gregg, permanent officers were elected as follows: For President, Dr. Bason, of Salsbury; Vice-president, Dr. Howlet, of Greensboro'; Secretary, Dr. Benbow, of Fayetteville; Treasurer, Dr. Bessent, of Concord; and Drs. Gregg, of Greensboro', Scott, of Washington and Harris, of Pittsboro', the Examining Committee.

On motion of Dr. Scott, Dr. Perry, of Raleigh, was unanimously elected a member of this Society.

Dr. Gregg offered the following resolution, which was adopted:

*Resolved,* That the President shall have the power to call a meeting of this Society whenever he shall consider the interests of the profession demand it, or whenever requested in writing to do so by five members.

It was then *Resolved,* That a committee of three be appointed to revise the Constitution and prepare By-Laws for our government, and report at our next meeting.

That committee consisted of Drs. Scott, Gregg and Perry.

On motion of Dr. Howlet, three members were elected to prepare and read papers at our next session, viz. Drs. Bason, Bessent and Gregg.

On motion of Dr. Benbow, it was *Resolved,* That when we adjourn we adjourn to meet in Greensboro' on the first Wednesday in October next.

On motion of Dr. Gregg, the President appointed Drs. Benbow, Bessent and Scott, to represent this Society in the next American Dental Convention to be held in Boston next August.

On motion of Dr. Scott, the President was added to the delegation.

The President hoped that other members of the Society might find it convenient to be there.

The Secretary was instructed to have published the proceedings of this Society.

After a few congratulatory remarks from Dr. Gregg, upon the kind feeling exhibited between the members of the profession, the meeting adjourned to meet in Greensboro' the first Wednesday in October, 1857.

W. F. Bason, *President.*

D. W. C. Benbow, *Secretary.*

### EDITORIAL DEPARTMENT.

